# PReS-FINAL-2089: Temporomandibular joint involvement and quality of life in juvenile idiopathic arthritis

**DOI:** 10.1186/1546-0096-11-S2-P101

**Published:** 2013-12-05

**Authors:** P Frid, E Nordal, F Bovis, D Marafon, D De Angelis, S Oliveira, G Simonini, F Corona, J Davidson, H Foster, R Joos, I Foeldvari, M Steenks, P Lahdenne, P Dolezalova, E Palmisani, A Martini, A Pistorio, N Ruperto

**Affiliations:** 1 Department of Otorhinolaryngology, Division of Oral and Maxillofacial Surgery, University Hospital North Norway and Public Dental Service Competence Centre of Northern Norway, Tromsø, Norway; 2 Department of Clinical Medicine, Faculty of Health Sciences, University of Tromsø and Department of Pediatrics, University Hospital of North Norway, Tromsø, Norway; 3 Istituto Giannina Gaslini, Pediatria II, Reumatologia, Paediatric Rheumatology International Trials Organisation (PRINTO) Coordinating Center, Genoa, Italy

## Introduction

Temporomandibular joint (TMJ) arthritis in childhood is seen in a substantial percentage of children with Juvenile idiopathic arthritis (JIA) and may lead to reduced mouth opening, pain and craniomandibular growth disturbances.

## Objectives

To assess the prevalence of TMJ involvement in a JIA cohort and the association between TMJ involvement and other disease variables such as cervical spine and upper limb involvement, and the impact of TMJ involvement on daily life.

## Methods

This descriptive study is based on data from a cross-sectional sample from 32 countries worldwide between 1998-2000, diagnosed with JIA, enrolled for validation of the Child Health Assessment Questionnaire (C-HAQ) and Child Health Questionnaire (CHQ), and also children enrolled in the PRINTO Methotrexate (MTX) trial. Disease activity and quality of life were assessed. Diagnosis of TMJ involvement was based on clinical assessment of the presence of swelling or limitation of motion (LOM) with pain and/or tenderness in at least one TMJ.

## Results

Of the 3344 children included, 68.3% were female and 45.8% were diagnosed with persistent or extended oligoarthritis. The prevalence of TMJ involvement was 11.6%. TMJ involvement was strongly associated with poly-arthritis (odds ratio (OR) 9.8 (confidence interval (CI) 6.1-15.8)), systemic (OR 7.4 (CI 4.5-12.3)) and extended oligo-arthritis (OR 6.7 (CI 4.0 - 11.1)) > 2 joints with LOM in upper limb > 2 (OR 9.8 (CI 7.5 - 12.7)) and cervical involvement (OR 7.8 (CI 6.2-9.8)) in univariate analysis. Finally, a multivariate logistic regression model with the disease activity measures, polyarticular JIA course, active joints >5, MTX use, female gender, age at visit, higher CHAQ scores, erythrocyte sedimentation rate (ESR), positive rheumatoid factor (RF) and other variables was performed; we underline the role of the following predictors in the association with the TMJ involvement: cervical spine involvement (3.0 (CI 2.2-4.0)), eating difficulties (1.2 (CI 1.0-1.4)) and superior limb involvement (1.1 (CI 1.0-1.1)).

## Conclusion

The prevalence of TMJ involvement was low in this large cohort compared with other studies probably due to a diagnosis based on clinical diagnostic criteria only. However, we found significant associations between TMJ involvement and cervical spine involvement, upper limb involvement, and eating difficulties. Further studies with both clinical and imaging diagnostic assessment of TMJ involvement in a longitudinal cohort study are warranted.

## Disclosure of interest

None declared.

